# Early oxytocin treatment in infants with Prader–Willi syndrome is safe and is associated with better endocrine, metabolic and behavioral outcomes

**DOI:** 10.1186/s13023-025-03560-3

**Published:** 2025-03-01

**Authors:** Marion Valette, Gwenaelle Diene, Mélanie Glattard, Julie Cortadellas, Catherine Molinas, Sandy Faye, Grégoire Benvegnu, Kader Boulanouar, Pierre Payoux, Jean-Pierre Salles, Catherine Arnaud, Sophie Çabal, Maithé Tauber

**Affiliations:** 1 Centre de Référence Maladies Rares PRADORT (syndrome de PRADer-Willi et autres Obésités Rares avec Troubles du Comportement Alimentaire), Hôpital des Enfants, CHU Toulouse, Université Toulouse III Paul Sabatier, 330, Avenue de Grande Bretagne, TSA 70034, 31059 Toulouse Cedex 9, France; 2 CERPOP (Centre d’Epidémiologie et de Recherche en sante des POpulations), UMR 1295 Inserm, Université Toulouse III Paul Sabatier, Toulouse, France; 3 Institut Toulousain des Maladies Infectieuses et Inflammatoires (Infinity) Inserm UMR1291 - CNRS UMR5051, Université Toulouse III Paul Sabatier, Toulouse, France; 4 Service Universitaire de Psychiatrie de l’Enfant et de l’Adolescent, CHU de Toulouse, Hôpital Purpan, Place du Dr Baylac, TSA 40031, 31059 Toulouse Cedex 9, France; 5 TOulouse NeuroImaging Center (TONIC), Université de Toulouse, Inserm UMR 1214, Université Toulouse III Paul Sabatier, Toulouse, France; 6 Unité d’épidémiologie clinique, CHU Toulouse, Toulouse, France

**Keywords:** Prader–Willi syndrome, Oxytocin, Infants, Long-term effects, Behavior, Metabolism, Brain connectivity

## Abstract

**Background:**

Oxytocin (OT) plays an important role in modulating behavior, social interactions and feeding. Prader–Willi syndrome (PWS), a rare genetic neurodevelopmental disorder, is a model of hypothalamic disorder including OT dysfunction. We previously showed that infants with PWS who had received an early short course (7 days) of intranasal OT treatment improved their oral and social skills. We aim to document the long-term tolerance and effects of early intranasal OT treatment on the disease trajectory.

**Methods:**

We performed a comparative clinical trial including the 17 children who had received OT as infants in our previous study and compared them to 17 PWS non-exposed children at 3–4 years old. Primary endpoint was the total communication score on the Vineland Adaptive Behavior Scales-2nd edition (VABS-II). Secondary endpoints were the other domains of VABS-II, behavior scored by the Child Behavior Checklist, feeding skills, endocrine and metabolic profiles, and brain connectivity on functional magnetic resonance imaging.

**Results:**

We documented the long-term safety of early OT treatment. The VABS-II communication score was not different between the two groups, defined as OT-exposed and non-exposed, whereas a trend toward a higher socialization score was found in the OT-exposed children (p = 0.06). Circulating IGF-1 and HDL cholesterol were significantly higher in the OT-exposed group (p < 0.05). OT-exposed children had normal acylated ghrelin levels, which were lower than those observed in non-exposed children (p = 0.06), and they displayed higher connectivity of the orbitofrontal cortex brain region.

**Conclusion:**

Early OT treatment in infants with PWS is safe up to 3–4 years of age. OT-exposed children display better social, endocrine and metabolic outcomes. This study documents for the first time in human the biological window of opportunity of early OT treatment, which may change the trajectory of the PWS condition.

*Trial Registration*: Clinical trial NCT03081832 Retrospectively registered https://clinicaltrials.gov/search?cond=NCT03081832.

**Supplementary Information:**

The online version contains supplementary material available at 10.1186/s13023-025-03560-3.

## Introduction

Oxytocin (OT) plays an important role in modulating social interactions, mother-infant bonding and feeding. A reduced number and volume of OT neurons in the paraventricular nucleus were reported in postmortem human hypothalamic tissue from patients with Prader–Willi syndrome (PWS) [1]. It is now acknowledged that the PWS phenotype is explained by impaired hypothalamic development and function, including the OT and ghrelin systems [2]. PWS is clinically characterized by a specific developmental trajectory involving neurodevelopmental, nutritional, endocrine and metabolic, and behavioral dimensions [2, 3]. PWS results from the loss of expression of paternally inherited imprinted genes of chromosome 15 at position q11–q13 due to a paternal microdeletion, a maternal uniparental disomy (mUPD), an imprinting defect or a translocation involving this region [2, 4, 5]. Nowadays, around 50% of newborns display a microdeletion. The non-deletion genotype subtype comprises newborns with mUPD (around 45%) and imprinting defect (around 5%).

Our previous phase 1/2 study, OTBB2 (NCT 02205034), showed that 16/18 infants with PWS younger than 6 months who had received a short course of intranasal OT for 7 days improved their oral and social skills, including mother-infant interactions, after the last OT administration [6]. Circulating acylated ghrelin (AG) levels and the connectivity of the right superior orbitofrontal network were significantly increased [6]. These results reproduced the preclinical data in *Magel2* knockout (KO) mice, a mouse model obtained after inactivating the *Magel2* gene, a maternally imprinted gene of the PWS chromosomal region, which showed that a single OT injection before the first 5 hours of life rescued 100% of the *Magel2* KO pups from early death by restoring normal sucking activity [7]. These rescued pups displayed normal socialization and increased memory and learning skills as adults [8].

In the current clinical trial, we evaluated the long-term tolerance and effects of 7 days of intranasal OT treatment in infants (< 6 months of age) who participated to the OTBB2 study by comparing them at 3–4 years of age to a group of age-matched children with PWS not treated with OT. This study aims to document for the first time in human the long-term tolerance of early OT treatment and whether it changes the course of the disease up to 4 years.

## Methods

### Study population

A detailed description of the population is shown in Table 1. Thirty-four children, 47% female and 41% with the deletion genetic subtype, were included in this comparative study. Children belonged to one of two groups, OT-exposed or non-exposed (17 children per group). These two groups were not statistically different regarding birth data, social status, family composition and schooling. In the whole group, the median age was 3.8 years (ranging from 3.0 to 4.3) with an interquartile range of 0.3, and the body mass index (BMI) Z-score was 0.08 (ranging from − 1.79 to 6.31). All children received growth hormone (GH) treatment. All children were followed in dedicated PWS centers except for one OT-exposed child due to extreme poverty of the family with high social deprivation score.

**Table 1:** Characteristics of the population

Variable	OT-exposed (n = 17)	Non-exposed (n = 17)	All (n = 34)
Age (yrs)	3.8 (3.1;4.2)	3.8 (3.0;4.3)	3.8 (3.0;4.3)
Sex Female n (%)	7 (41%)	9 (53%)	16 (47%)
*Genetics*			
Deletion n (%)	6 (35%)	8 (47%)	14 (41%)
Non-deletion n (%)	11 (65%)	9 (53%)	20 (59%)
mUPD n (%)	9 (53%)	9 (53%)	18 (53%)
Imprinting defect n (%)	2 (12%)	0	2 (6%)
*Auxological data—body composition*			
Weight (kg)	15.4 (12.2;28.4)	14.6 (13;19.6)	15.2 (12.2;28.4)
Weight (SDS)	0.7 (− 1;10,7)	0.6 (− 1.1;4.8)	0.6 (− 1.1;10.7)
Height (cm)	98.5 (92.5;108)	96 (90.5;107)	97.9 (90.5;108)
Height (SDS)	0.8 (− 1.2;3.9)	0.5 (− 1.4;4.3)	0.6 (− 1.4;4.3)
BMI kg/m^2^	15.9 (14.1;26.3)	15.4 (13.8;21.7)	15.8 (13.8;26.3)
BMI (Z-score)	0.13 (− 1.5;6.31)	− 0.35 (− 1.79;4.23)	0.08 (− 1.79;6.31)
BMI Z-score ≥ 2	2 (12%)	2 (12%)	4 (12%)
Head circumference (cm)	49 (46.5;53) (n = 16)	50 (48;52.5) (n = 17)	50 (46.5;53) (n = 33)
Head circumference (SDS)	− 0.35 (− 2.5;2.7) (n = 16)	− 0.31 (− 1.9;1.25) (n = 17)	− 0.31 (− 2.5;2.7) (n = 33)
Body fat mass (%)	22 (10;65) (n = 17)	24.5 (8;40) (n = 16)	22 (8;65) (n = 33)
Body lean mass (%)	75 (32;85) (n = 17)	71 (57;88) (n = 16)	73 (32;88) (n = 33)
*GH treatment*			
% of treated	100%	100%	100%
Dose at start GH treatment (µg/kg/day)	26 (15;41)	25 (8;52)	26 (8;52)
Current GH dose (µg/kg/day)	27.6 (15.2;41.4)	28.6 (6.8;51.5)	28.1 (6.8;51.5)
Age at start GH treatment (months)	10.0 (7.2;12.4)	13.2 (7.5;30.3)	10.6 (7.2;–30.3)
% with at least 2 years of treatment	100%	94%	97%
*Psychosocial and family data*			
Number of siblings	0 (0;2)	1 (0;3)	1 (0;3)
Couple n (%)	12 (71%)	14 (82%)	26 (77%)
Schooling n (%)	12 (71%)	14 (82%)	26 (77%)
Social vulnerability score (EPICES)*	16.6 (0;100) (n = 17)	16.9 (0;73) (n = 16)	16.6 (0;100) (n = 33)
*Birth data*			
Term (WA)	39 (30;41)	39 (32;41)	39 (30;41)
Premature birth n (%)	3 (18%)	3 (18%)	6 (18%)
Cesarean delivery n (%)	9 (53%) (n = 17)	10 (59%) (n = 16)	19 (56%) (n = 33)
Weight (SDS)	− 1.7 (− 2.9;− 0.3) (n = 17)	− 1.7 (− 2.3;0) (n = 16)	− 1.7 (− 2.3;0) (n = 33)
Length (SDS)	− 0.9 (− 2.5;0) (n = 17)	− 1.0 (− 2.6;0) n = 16)	− 0.9 (− 2.6;0) (n = 33)
Nasogastric tube feeding (NGT) n (%)	15 (88%)	13 (76%)	28 (82%)
Duration of NGT (days)	120 (14;195)	31 (2;210)	85 (2;210)

Results are presented as n (%) or median (min;max) by groups and in all children. SDS = standard deviation score. There was no statistical difference between the two groups regarding any of the parameters in the table, including median duration of NGT (p = 0.15)

^*^EPICES questionnaire: The maximum score is 100, and the cutoff of 30 was used to define social deprivation

### Study protocol

This single-center clinical trial compared the adaptive functioning, behavior and development, feeding and social skills, endocrine and metabolic profiles, and safety of the 3- to 4-year-old PWS children who composed the two groups mentioned above. The OT-exposed group comprised 17 children who had participated as infants in the OTBB2 study. The OTBB2 study was an open-label phase 1/2 dose-escalation study with three groups of six infants with PWS, each group receiving the same dose that increased from group 1 to group 3 as follows: OT 4 IU every other day, every day, or twice a day for a total duration of treatment of 7 days. Children of the non-exposed group were aged-matched (with a maximum difference of ± 3 months) with children of the OT-exposed. All children were admitted for a single 3-day visit to the hospital hosting the French reference center for PWS in Toulouse. The complete study protocol is available at https://www.chu-toulouse.fr/IMG/pdf/final_protocole__ot2suite_v1.6_22032017-en.pdf and details of inclusion and exclusion criteria are detailed below.

#### Inclusion/exclusion criteria

Among the 18 patients included in the OTBB2 study, one was lost to follow-up; the 17 remaining patients were included in this current study. One patient combining severe social economic deprivation regarding the EPICES (Evaluation of the Deprivation and Inequalities of Health in Healthcare Centers) score (> 50) and poverty, defined as extreme deprived conditions of life confirmed by the clinical team, was excluded from the VABS-II socialization domain analysis because both conditions have a severe impact on care, social adaptive skills and behavior.

#### Outcomes and data collected

*Adaptive functioning*: The primary endpoint was the score in the communication domain of the VABS-II [9]. The VABS-II evaluates adaptive functioning in four domains: communication, socialization, daily living skills and motor skills expressed in scores and categorized as adequate, moderately low or low. Age equivalent scores and standard scores [mean (M) = 100; standard deviation scores (SDS) = 15] are provided for each domain. The other subscores of the VABS-II were analyzed as secondary endpoints as were the following evaluations.

*Behavior*: Behavior was assessed with the Child Behavior Checklist (CBCL) using its total T score and 14 subscores [10].

*Feeding skills*: A dynamic videofluoroscopy of swallowing (VFSS) was performed and scored with a chart including the analysis of the five phases of swallowing. Eating behavior was assessed by a questionnaire used in routine care in our center. It was completed by the pediatrician during an interview with the parents, with 5-point Likert scale responses (Supplementary Table 1).

*Growth and development*: Children were measured and weighed during the study visit. Development was assessed by the Bayley Scale of Infant and Toddler Development version III (BSID-III), which included cognitive, receptive language, expressive language, fine motor, and gross motor scores [11].

*Endocrine and metabolic evaluations*: Sampling for hormone and metabolic assays: measurements of Insulin-like Growth Factor 1 (IGF-1), thyroid hormones, glycemia, insulin and lipids were performed as routine evaluations. Plasma concentrations of acylated ghrelin (AG), unacylated ghrelin (UAG) and OT were measured as previously described [6]. We then compared the ghrelin data with data from age-matched healthy controls from our previous studies [12, 13].

*Brain connectivity analysis during resting state using functional MRI (fMRI)*: The children lay supine and were instructed to relax and keep their eyes closed, but not to sleep. fMRI preprocessing and statistical analyses are described in the supplementary methods. We chose to study the default mode network (DMN), which has frequently been studied, and the orbitofrontal cortex network (superior, median and inferior) as in the OTBB2 study [6].

To reveal the areas where significant connectivity change occurred, we set up the threshold for the statistical maps at p < 0.005 voxel-wise, and we used a threshold for cluster extent of p < 0.05 corrected for multiple comparisons across the whole brain.

*Comorbidities*: Sleep disorders and orthopedic problems reported in the children’s medical files were collected.

*Evaluation of tolerance*: Tolerance was assessed by recording vital signs, physical examination signs, laboratory parameters and adverse events (AE).

*Evaluation of social deprivation*: The EPICES score is a self-questionnaire validated in France [14, 15] that evaluates the social deprivation of the family as defined by limited access to society’s resources due to poverty, discrimination, or other disadvantages. The score ranges from 0 to 100 with a threshold of > 30 defining social deprivation.

### Statistical analysis

Comparisons between the OT-exposed and non-exposed groups were performed.

Results for all variables, including each item of the questionnaires, were summarized by group using descriptive statistics or frequency/percentage, as appropriate. A parametric approach (Student t-test) or a non-parametric approach (Mann-Whitney)—according to the normality/non-normality distribution—was used to compare groups. Subgroup statistical analysis was performed using an analysis of variance (ANOVA). The model included group (treated vs. untreated) and age group (1st, 2nd, and 3rd terciles) as fixed effects.

Subgroup analysis was performed to explore the group effect within two levels of genetic diagnosis (deletion and non-deletion). When differences were found between the two genetic subtypes within the OT-exposed versus non-exposed groups, they are mentioned in the Results section. Differences in the proportions of normal and abnormal patients between the OT-exposed and non-exposed groups, as defined by thresholds, were analyzed using a Cochran–Mantel–Haenszel test with age group as the controlling factor.

Statistical analysis was performed using SAS® Enterprise Guide software, version 7.1 (SAS Institute, Cary, NC, US) and Stata version 11.2 (Stata Corp, College Station, TX, US). The statistical significance threshold was set at 0.05.

## Results

### Adaptive behavior

#### Primary endpoint: VABS-II *communication domain*

**Fig. 1:**
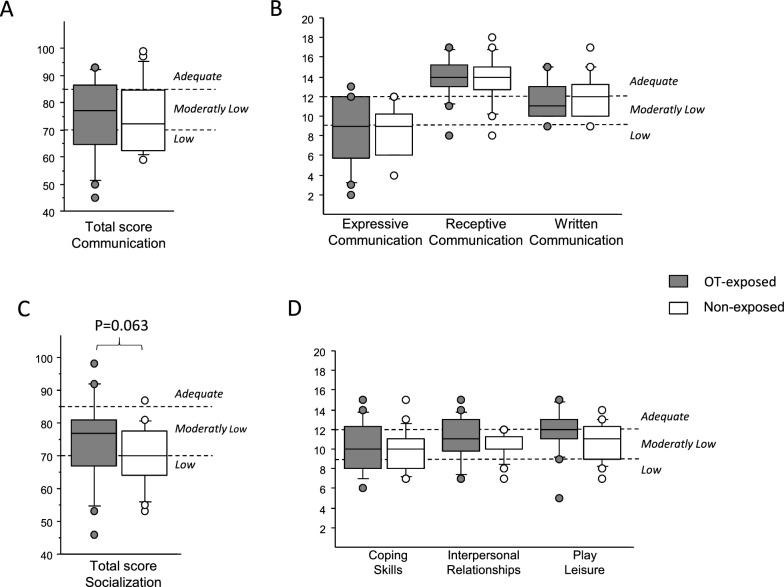
VABS II total scores and subscores of communication and socialization domains. VABS total scores and subscores of communication (**A**, **B**) and socialization (**C**, **D**) in OT-exposed (gray) and non-exposed (white) groups. Horizontal dotted lines represent the thresholds above which scores were considered as low, moderately low and adequate scores

The mean total communication score (74 in OT-exposed vs. 75 in non-exposed) and subscores were not significantly different between the two groups (Fig. 1A, B).

#### Secondary endpoints

##### Socialization domain

This domain comprises three subscores: coping skills, interpersonal relationships and play leisure. We found a trend for higher mean total socialization score in the OT-exposed group, excluding the child with social deprivation and extreme poverty (76 in OT-exposed vs. 70 in non-exposed, p = 0.063), adjusted for age (Fig. 1C, D) compared to the non-exposed group. The total socialization score was adequate in 24% versus 6% in the OT-exposed versus non-exposed group (p = 0.098). In the non-deletion genetic subtype groups, the interpersonal relationships subscore was adequate in 29% in the OT-exposed children versus none in the non-exposed children (p = 0.020).

##### Other domains of the VABS-II

The two other domains of the VABS-II (daily living skills and motor skills) were not significantly different between the OT-exposed and non-exposed groups, whether using total scores or subscores.

### Behavior

**Fig. 2:**
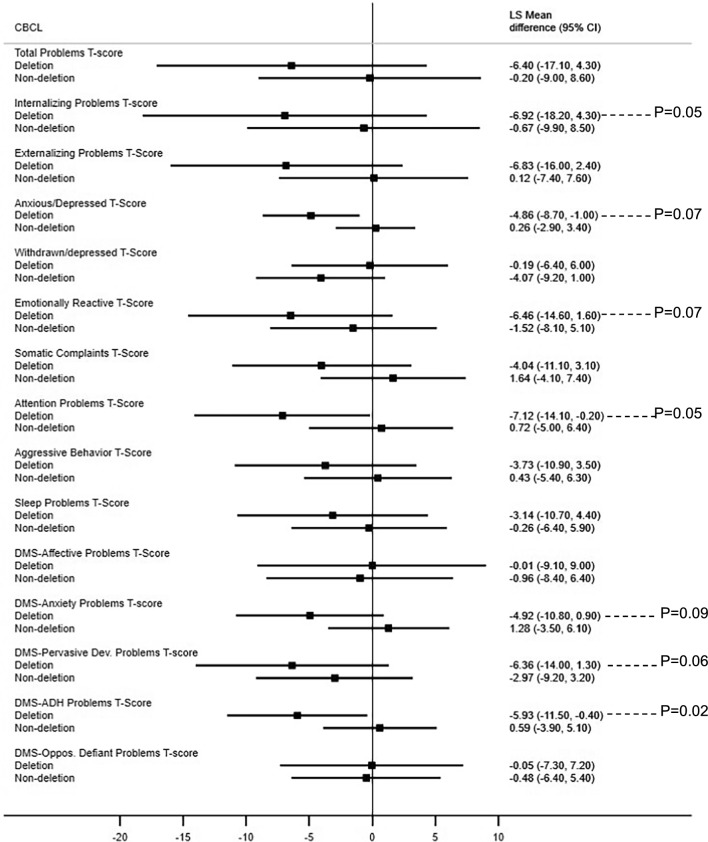
CBCL subscores in the OT-exposed and non-exposed groups according to genetic subtype groups (deletion vs. non-deletion). This figure shows the forest plot comparing the difference between the two genetic subtype groups (deletion vs. non-deletion) within the OT-exposed and non-exposed groups for each subscore. In the deletion subtype group, there were significant differences or trends between the OT-exposed group and the non-exposed group in subscores adjusted for age

The mean total score of the CBCL was not significantly different between the two groups when adjusted for age (52 in the OT-exposed vs. 55 in the non-exposed group, p = 0.37). The internalizing problems subscore was normal in 94% versus 65% (p = 0.04) and the emotionally reactive subscore was normal in 94% versus 71% (p = 0.08) in the OT-exposed versus the non-exposed group, respectively. Figure 2 shows the forest plot comparing the difference between the deletion versus non-deletion genetic subtype groups within the OT-exposed and the non-exposed groups. In the deletion subtype group, we found statistically significant differences or trends in many subscores between the OT-exposed and non-exposed group, with better subscores in the OT-exposed children (Fig. 2).

### Oral skills

The total VFSS score was abnormal in all children. No statistically significant difference was observed between the OT-exposed and non-exposed groups for the mean total score or the distribution of children with normal and abnormal subscores, although there were trends for more normal subscores in the OT-exposed group (see Supplementary Table 2).

### Feeding behavior

Mean total score of the feeding behavior questionnaire was similar in the OT-exposed and non-exposed groups (11.6 vs. 13.3, p = 0.5). We found a significant difference only for question 13 in the non-deletion genetic subtype groups (interaction treatment*genotype, p = 0.02) with 55% of the non-exposed children requiring food access control versus no children in the OT-exposed group.

### Development

The BSID-III scores were not different between the two groups. Most of the children were classified as abnormal (see Supplementary Figure 1).

### Comorbidities

Comorbidities are listed in Table 2.

**Table 2:** Comorbidities presented by the children in each group and comparison between groups

Variable	OT-exposed (n = 17)	Non-exposed (n = 17)	p-value
*Endocrine and metabolic comorbidities*			
IGF-1(ng/mL)	**198 (36;267)**	**132 (26;266) (N = 16)**	**0.01**
Free thyroxine (ng/L)	13.2 (10.4;15)	12.2 (9.5;15.3)	0.07
Treatment by L-thyroxine n (%)	10 (59%)	5 (29%)	NS
Dosing (µg/d)	17.5 (10;50)	20 (10;25)	NS
HDL cholesterol (g/L)*	**0.52 (0.38;0.82)**	**0.46 (0.33;0.6)**	**0.02**
LDL cholesterol (g/L)	1.05 (0.67;1.95)	1 (0.36;1.8)	NS
VLDL cholesterol (g/L)	0.15 (0.08;0.22)	0.14 (0.1;0.28)	NS
Total cholesterol (g/L)	1.71 (1.27;2.59)	1.61 (1.02;3.48)	NS
Triglycerides (g/L)	0.73 (0.39;1.12)	0.7 (0.5;1.4)	NS
Glycemia (g/L)	0.76 (0.6;1.08)	0.74 (0.52;0.9)	NS
Insulinemia (mIU/L)	5.1 (1.9;18.3) (N = 15)	4.8 (1.9;12.2) (N = 15)	NS
HOMA-IR^£^ (min–max)	0.9 (0.4;4.9)	1 (0.3;2.3)	NS
*Sleep respiratory disorders*			
(polysomnography before GH)	N = 13	N = 12	
Apnea hypopnea index (AHI)	2.9 (0.5;15.5)	3.5 (0;56.8)	NS
Obstructive apnea index (OAI)	0 (0;9.9)	0 (0;2.3)	NS
Central apnea index (CAI)*	0.5 (0;4.4) (N = 13)	2.3 (0;18.7) (N = 12)	0.06
% of children with ICA ≥ 1	**46%**	**83%**	**0.03**
*Scoliosis* n (%)	6 (35%)	5 (29%)	NS
Age at diagnosis (yrs)	1.75 (1.05;2.11)	1.69 (1.09;2.19)	
Cobb angle (°)	30 (15;56) (N = 5)	29 (17;80) (N = 3)	

Results are presented as n (%) or median (min;max) by groups and in all children

Bold values are statitically different between the 2 groups, OT-exposed and non-exposed

*Logistical regression adjusted for age. ^£^ Homeostatic Model Assessment for Insulin Resistance

*Endocrine and metabolic disorders*: All children received GH treatment at the same recommended dose, and median IGF-1 levels were in the normal range with significantly higher values in the OT-exposed group compared to the non-exposed group (198 ng/mL vs. 132 ng/mL, p = 0.01). Median HDL-cholesterol levels were significantly higher in the OT-exposed group compared to the non-exposed group (0.52 g/L vs. 0.46 g/L, p = 0.02). There was a trend for higher free T4 in the OT-exposed group, which was related to the higher frequency of L-thyroxine treatment in this group due to the fact that treatment was started by the reference centre after the inclusion of infants in OTBB2 study.

**Fig. 3:**
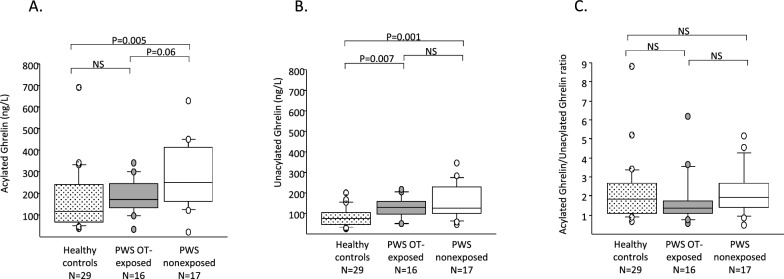
Circulating AG (**A**), UAG (**B**) and AG/UAG ratio (**C**) levels in OT-exposed (gray box) and non-exposed (white box) PWS children and age-matched healthy controls (dotted box). The healthy control group comprises 29 children, mean age 3.5 years, from a previous study [12]

*Ghrelin levels*: The OT-exposed group showed a trend for lower AG levels versus the non-exposed group (172 ng/mL vs. 257 ng/mL, p = 0.06) with no significant difference in UAG and a trend for a lower AG/UAG ratio (1.37 vs. 1.91, p = 0.09) (Fig. 3). When compared to a healthy control group of the same age range, the OT-exposed group had similar AG levels (172 ng/mL in OT-exposed group vs. 115 ng/mL in healthy controls), whereas UAG was significantly higher (130 ng/mL vs. 76 ng/mL*,* p = 0.007) with no difference in the AG/UAG ratio. The non-exposed group had significantly higher levels of both AG (257 ng/mL vs. 115 ng/mL, p = 0.03) and UAG (127 ng/mL vs. 76 ng/mL*,* p=0.005) compared to the healthy control group adjusted for age with no difference in the AG/UAG ratio.

*Sleep disorders*: Patients underwent polysomnography before the start of GH treatment at a median age of 8.5 months (range 5.1–10.7 months) in the OT-exposed group (n = 13) versus 12.6 months (range 7.3–26) in the non-exposed group (n = 12). Forty-six percent of OT-exposed children had central apnea [central apnea index (CAI) > 1] before initiating GH treatment versus 83% of the non-exposed children (p = 0.03). We found a trend toward a lower median CAI adjusted for age in the OT-exposed group versus the non-exposed group (0.5 vs. 2.3/h, respectively, p = 0.06). No difference was observed after GH treatment.

*Scoliosis*: No difference was observed regarding the occurrence of scoliosis: 35% in the OT-exposed group versus 29% in the non-exposed group.

### Safety

Overall, three subjects in the OT-exposed group (17.6%) and seven subjects in the non-exposed group (41.2%) reported at least one adverse event during the study (see Supplementary Table 3), none of them related to the intranasal OT treatment.

Comparison of body measures, vital signs, and standard laboratory parameters between the OT-exposed and the non-exposed groups showed no evidence of any negative effect of OT treatment.

### Circulating OT levels

No difference in circulating OT levels was found between the two groups: 0.44 ng/L in the OT-exposed group versus 0.45 ng/L in the non-exposed group.

### Brain fMRI

**Fig. 4:**
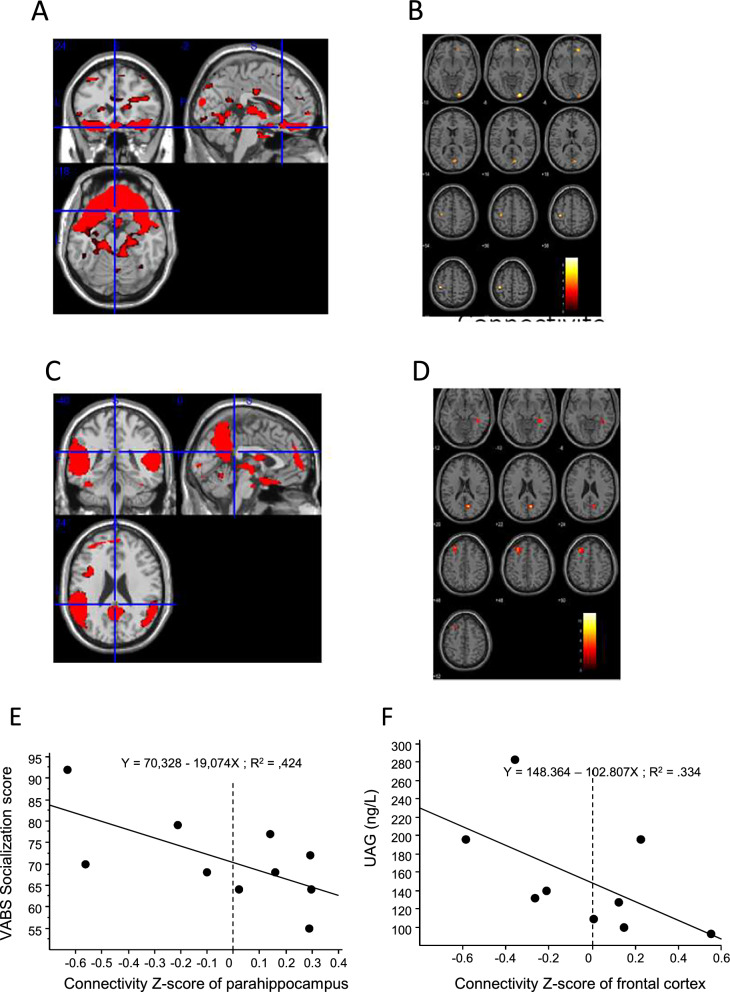
Brain connectivity studies in fMRI in the OT-exposed and non-exposed groups. Brain region connectivity in the orbitofrontal network (**A**, **B**) and default mode network (DMN) (**C**, **D**) are shown. High connectivity regions in each network are shown in red (**A**, **C**). **B** and **D** showed higher connectivity Z-score in the OT-exposed children compared to non-exposed children in red (**B**) and in yellow (**D**); Correlations between connectivity Z-score of parahippocampus and VABS-II socialization score (**E**). Correlations between connectivity Z-score of frontal cortex and UAG level in DMN (**F**)

Among the 18 patients who underwent fMRI, only 10 (6 in the OT-exposed group and 4 in the non-exposed group) were able to stay motionless during fMRI acquisition and completed the whole experiment. In the DMN analysis, the OT-exposed group showed higher connectivity of the right hippocampus, precuneus and the medial frontal cortex regions (p < 0.005) (Fig. 4A, C). In the orbitofrontal component analysis, the OT-exposed group showed a higher connectivity in the inferior occipital, precentral, medial frontal and cuneus regions (p < 0.005) (Fig. 4B, D). There was a negative correlation within the whole group between the VABS-II total score of socialization and the connectivity of the parahippocampus (p = 0.036) (Fig. 4E) and a negative correlation between the circulating UAG level and the connectivity of the frontal cortex (p = 0.022) (Fig. 4F).

## Discussion

This clinical trial allowed us to document for the first time the good long-term tolerance of early OT treatment of infants with PWS who participated to the OTBB2 study [6] and to compare the characteristics and severity of the disease in these children at 3–4 years with an age-matched non-exposed group of PWS children. We did not find a statistically significant difference between the two OT-exposed and non-exposed groups for adaptive skills of the communication domain using the VABS-II, which was the primary endpoint of our study. We found a strong signal regarding the socialization domain, with a trend toward higher scores and more children with adequate socialization scores in the OT-exposed group compared to the non-exposed group. This long-term effect of OT on social skills was demonstrated in the *Magel2* gene inactivated mouse model and is consistent with the known effect of OT on bonding and on setting the network for social recognition [16].

Regarding the comprehensive evaluation of behavior using the CBCL, which is widely used in PWS, we did not find a significant difference between the OT-exposed and non-exposed groups. Interestingly, in the deletion genetic subtype group we found better outcomes in the OT-exposed versus non-exposed children in most of the subscores. The influence of genetic subtype on the effect of OT treatment was also reported in a 3-month study in children with PWS (age 3–11 years) [17]. This study showed significant improvement in Dykens’ Hyperphagia questionnaire [18] and the global behavior score after OT treatment versus placebo only in the deletion group of children [17]. Conversely, in our study, the differences between the OT-exposed and non-exposed groups regarding the need for control access for food was only observed in the non-deletion genetic subtype children. For the other endpoints, we did not find an influence of the genetic subtype. Oral skills analyzed by VFSS were not significantly different between the two groups, but we observed a trend for more frequently adequate swallowing pharyngeal initiation and protection of respiratory airways in the OT-exposed group.

Interestingly, we found significant differences between the OT-exposed and non-exposed groups regarding endocrine and metabolic comorbidities. Although all children received GH treatment at the same dose, the OT-exposed group displayed significantly higher levels of IGF-1. This suggests greater GH sensitivity, which may be explained by a better metabolic outcome as HDL-cholesterol levels were also significantly higher in the OT-exposed group. Hyperghrelinemia is a cardinal metabolic marker of PWS. As a group, patients with PWS display high AG and UAG levels at all ages [19]. We previously showed normalization of AG levels in the OTBB2 trial [6], and we report in this study that this effect persisted up to 4 years of age. The results suggest that early OT treatment may prevent the switch in the ghrelin system observed in PWS [12, 19]. The significant differences observed in endocrine and metabolic trajectories are unexplained but are comparable to the results obtained by attenuating the leptin effect in early life, which improves lifelong metabolic regulation in postnatally overnourished mice [20].

Central apnea is a common feature in infants and children with PWS [21]. Our study showed that the OT-exposed children displayed central apnea less frequently before the start of GH treatment than the non-exposed children, which suggests that early OT treatment may also prime the autonomic system linked with respiratory control. One study reported a positive effect of OT administration on sleep respiratory disorders in non-PWS adult obese patients and showed a reduction in the frequency of hypopneas and the duration of apneas and hypopneas [22].

The current study showed differences between the OT-exposed group and the non-exposed group in the brain connectivity of regions involved in neurodevelopment which were identified in the OTBB2 study [6]. These findings therefore document a long-term effect of OT on brain plasticity, particularly in regions involved in the regulation of socialization and feeding, such as the parahippocampus and the frontal cortex [23, 24]. As a whole, our study paves the way to demonstrate the long-term effects of early OT treatment during a critical postnatal period in humans [25]. The children of the OT-exposed group are now 9–10 years old. They are routinely followed in our center, and we confirm excellent long-term tolerance of early OT treatment (data not shown).

We recently completed a phase 3 European study comprising 52 PWS infants who received a longer course of OT treatment (1 or 2 months). These OT-treated infants will be followed yearly until 4 years of age to confirm the good long-term tolerance and will be compared to non-exposed children in order to confirm our current data with a longer duration of early OT exposure of infants.

Limitations and strengths of the study: the study population was small due to the rarity of the disease, and this may explain why the differences in outcomes were not significant but rather trends for better outcomes in the exposed group. The strength of this study is that evaluations including biological and endocrine aspects were performed in the same expert center with patients coming from the entire country.

In conclusion, our comparative study demonstrated the long-term safety of an early short course (7 days) of intranasal OT treatment in infants with PWS and the better outcomes or trends at 3–4 years regarding behavioral, endocrine and metabolic profile, as well as differences in the connectivity of brain regions of interest. This study documents for the first time in human the critical postnatal period that offers a window of opportunity to implement OT treatment to change the trajectories of the disease.

## Supplementary Information

Additional file 1

## Data Availability

The data that support the findings of this study are available from the CHU of Toulouse but restrictions apply to the availability of these data, which were used under license for the current study, and thus are not publicly available. Data are, however, available from the authors upon reasonable request and with permission of the CHU of Toulouse.
