# Supplementary material for: Early oxytocin treatment in infants with Prader–Willi syndrome is safe and is associated with better endocrine, metabolic and behavioral outcomes
Source: Orphanet J Rare Dis. 2025 Mar 1;20:96. doi: 10.1186/s13023-025-03560-3 (PMC11872305; doi:10.1186/s13023-025-03560-3)
Supplement: Supplementary file 1 — Additional file 1 [file 13023_2025_3560_MOESM1_ESM.docx]

**Supplementary Data**

Index

Supplementary Table 2: Eating questionnaire2

Supplementary Table 3: Normal and abnormal sub score of the VFSS in the two groups, OT-exposed and nonexposed 3

Supplementary Table 4: Summary of Adverse Events by System Organ Class and Preferred Term4

Supplementary Figure 1: Development assessment with BSIDIII5

Supplementary Method: MRI analyses6

# **Supplementary Table 1: Eating questionnaire.**

This eating questionnaire is routinely used in our center. Each of the 13 questions was scored from 0 to 5 points using a Likert scale (from 0-completely disagree to 5-completely agree); the total score is the sum of the answers to the 13 items of the table.

**EATING BEHAVIOUR EVALUATION QUESTIONNAIRE**

Child: ………………………………..

Date: ………………… Age: …………………

Evaluation performed by :  mother  father

|  | **Completely disagree** | **Disagree** | **Neither agree, nor disagree** | **Agree** | **Completely agree** |
| --- | --- | --- | --- | --- | --- |
| 1. Your child eats excessively (for his/her age) 🡪 Since when has his/her appetite been increased? ………………………………………………. |  |  |  |  |  |
| 2. He/she is excessively concerned about food |  |  |  |  |  |
| 3. He/she asks for large portions when served |  |  |  |  |  |
| 4. He/she puts too much food in his/her mouth at once |  |  |  |  |  |
| 5. He/she compares what's on his/her plate with others |  |  |  |  |  |
| 6. He/she always leaves a clean plate |  |  |  |  |  |
| 7. He/she tends to want to finish other peoples' meals |  |  |  |  |  |
| 8. He/she is hungry immediately after leaving the table |  |  |  |  |  |
| 9. He/she eats between meals |  |  |  |  |  |
| 10. He/she eats in secret |  |  |  |  |  |
| 11. He/she tends to make hidden stores of food or regularly steals food |  |  |  |  |  |
| 12. He/she eats unusual things or food unfit for human consumption.  Description : |  |  |  |  |  |
| 13. Is food access controlled:  Yes /  No  Methods:  don't leave food lying around in view  lock the fridge  lock food away in cupboards  other: |  |  |  |  |  |

14. Time of day when he/she is most hungry:

morning  lunchtime  tea-time evening  no difference noted

15. Please provide an open description of your child's behavior:

# **Supplementary Table 2:** Normal and abnormal sub score of the VFSS in OT-exposed and nonexposed.

There was a trend for higher percentage of normal swallowing initiation and synchronization sub score (29.4% vs. 6.3%, p=0.066) and pharyngeal phase and protection of respiratory airways (88.2% vs. 62.5%, p=0.08) in the OT-exposed vs. nonexposed group.

|  | **OT-exposed (N=17)** | | **Nonexposed (N=17)** | | **P-value^1^** |
| --- | --- | --- | --- | --- | --- |
|  | **Normal**  **n (%)** | **Abnormal**  **n (%)** | **Normal**  **n (%)** | **Abnormal**  **n (%)** |  |
| Oral Phase | 3 (17.6) | 14 (82.4) | 6 (37.5) | 10 (62.5) | 0.234 |
| Oral and pharyngeal propulsion | 0 | 17 (100) | 1 (6.3) | 15 (93.8) | 0.2482 |
| Swallowing Initiation and synchronization | 5 (29.4) | 12 (70.6) | 1 (6.3) | 15 (93.8) | 0.0666 |
| Pharyngeal Phase and protection of respiratory airway | 15 (88.2) | 2 (11.8) | 10 (62.5) | 6 (37.5)0 | 0.0899 |
| Esophageal Phase | 4 (23.5) | 13 (76.5) | 4 (25) | 12 (75) | 0.9522 |

^1^ p-value from a Cochran-Mantel-Haenszel (CMH) test with age group (1st, 2nd and 3rd terciles) as controlling factor, comparing the proportion of Normal/Abnormal between groups.

# **Supplementary Table 3:** summary of adverse events by System Organ Class and Preferred Term

| **System Organ Class**  **Preferred term** | **OT-exposed**  **(N=17)**  **n (%)** | **Nonexposed**  **(N=17)**  **n (%)** |
| --- | --- | --- |
| **Subjects with at least one AE** | **3 (17.6%)** | **7 (41.2%)** |
| **Infections & Infestations**  Paronychia | 0  0 | 1 (5.9%)  1 (5.9%) |
| **Investigations**  Blood Thyroid Stimulating Hormone increased  Dehydroepiandrosterone increased  High Density Lipoprotein Decreased  Leptin Level Increased  Lipoprotein (A) Increased | **1 (5.9%)**  0  0  0  1 (5.9%)  0 | **4 (23.5%)**  1 (5.9%)  1 (5.9%)  1 (5.9%)  0  1 (5.9%) |
| **Metabolism & Nutrition Disorders**  Hypercholesterolemia  Hyperinsulinism  Insulin Resistance  Vitamin Deficiency | **2 (11.8%)**  1 (5.9%)  2(11.8%)  1 (5.9%)  1 (5.9%) | **1 (5.9%)**  1 (5.9%)  0  0  0 |
| **Nervous System Disorders**  Cerebral Cyst | **1 (5.9%)**  1 (5.9%) | **0**  0 |
| **Respiratory, Thoracic & Mediastinal Disorders**  Sleep Apnea Syndrome  Asthma | **1 (5.9%)**  1 (5.9%)  0 | **2 (11.8%)**  1 (5.9%)  1 (5.9%) |
| **Skin & Subcutaneous Tissue Disorders**  Dry skin  Eczema  Pruritus | **1 (5.9%)**  0  1 (5.9%)  0 | **2 (11.8%)**  2 (11.8%)  0  1 (5.9%) |

AE: Adverse Event

Note (s): Subjects experiencing multiple AEs within the same system organ class (SOC) were counted only once for that SOC. Similarly, subjects experiencing multiple AEs within the same preferred term (PT) were counted only once within that PT.

Adverse events are coded using MedDRA version 20.0.

# **Supplementary Figure 1:** Development assessment with BSID-III.

Sub scores of cognitive, expressive communication, fine motor, gross motor, receptive communication (in treated (blue) and untreated (red) groups. Scores were expressed in quotient of development (QD) i.e. QD = (developmental age/chronological age) *100. QD< 80 requires care and support

#
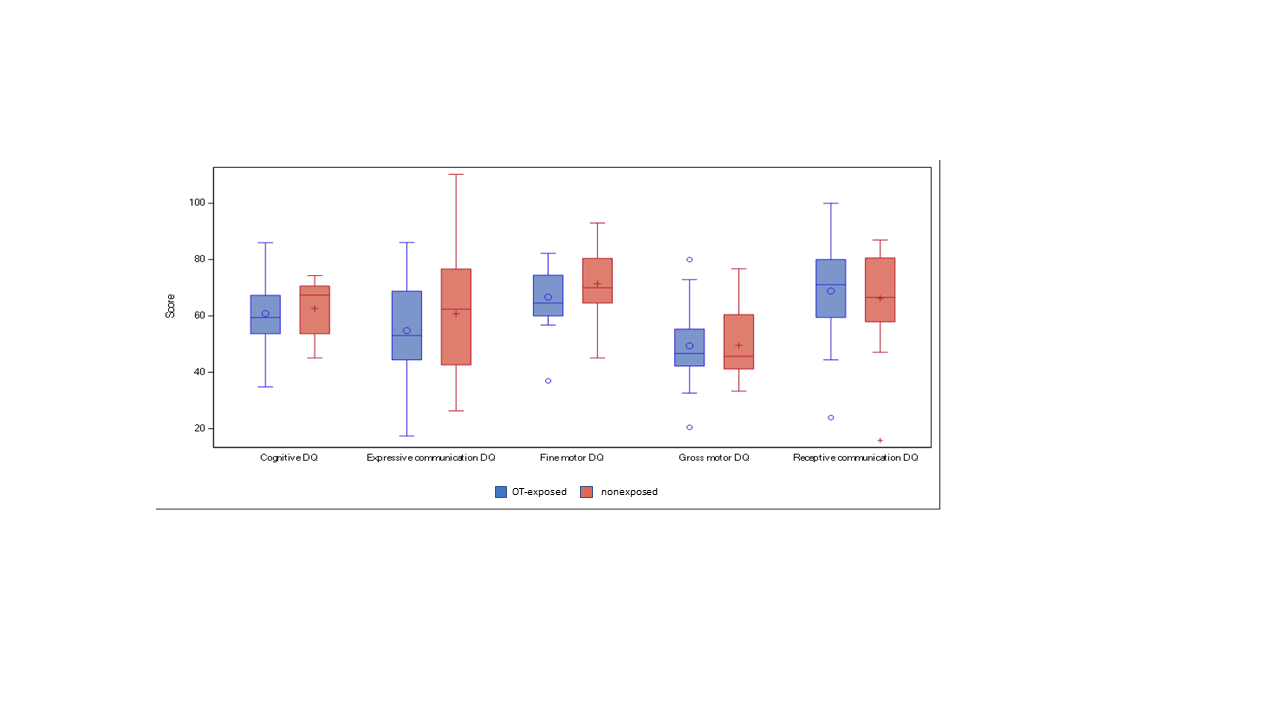


# Supplementary Method: fMRI analyses

We used a 3.0T MRI scanner (Achieva, Philips Medical Systems, Amsterdam, The Netherlands) equipped with a whole head SENSE coil. Field-echo echo-planar imaging (FEEPI) pulse sequence was used in the acquisition of T2*-weighted functional MR images of blood oxygenation level-dependent (BOLD) contrast. The FEEPI protocol was defined according to the following parameters: time to echo (TE) = 33 ms, time to repetition (TR) = 2800 ms, flip angle = 90°, field of view = 240x240x105 mm, matrix = 80x80x35, in plane voxel size = 2.9x2.9 mm. The images were acquired in 35 interleaved ascending slices (3-mm thickness) in a transverse plane. For each run, 220 resting state images were acquired. T1-weighted 3D images were acquired for registration and spatial normalization of the functional images as well as for anatomical localization. The T1-weighted, 3D images were acquired using the T1W_IR (Inversion Recovery) Philips sequence with TR= 5007 ms TE=13 ms; IT=400 ms; in plane pixel spacing 1x1 mm; slice spacing: 1.5 mm; flip angle 90° matrix 240x240x150. The acquisition time of the entire protocol was about 30 min.

*fMRI data Pre-processing*

Data pre-processing of imaging data were performed using the Statistical Parametric Mapping package^2^ for MATLAB, SPM12 (www.fil.ion.ucl.ac.uk) and MRIcron software^3^. To allow group analysis, we normalized the brain in the functional images on the same template, the Montreal Neurological Institute template, using anatomical images (T1-weighted). First, all volumes of the fMRI dataset were rigidly registered to the first volume of the same run. The differences in translational and rotational head motion, for all subjects and all acquisitions did not exceed 3 mm in translation or 1 degree in rotation. Slice-timing correction was then performed on the motion-corrected data. To achieve atlas registration, we aligned the fMRI image to the anatomic T1 image by computing an affine transformation. The T1 image was then registered to MNI152 brain template of the Montreal Neurological Institute using Normalised Mutual Information Coregistration as implemented in SPM software. The transformations were then concatenated and used to resample the fMRI image to the MNI atlas space with a 2-mm isotropic voxel resolution. The fMRI data were then spatially smoothed by using a Gaussian kernel of 8-mm full width at half maximum.

*Resting-State image processing and statistical data analysis*

To assess brain connectivity, we used group ICA4 as implemented in GIFT5 by introducing all the subjects and the three acquisition per subject into a single ICA analysis. Compared to individual analysis, the group analysis has the advantage of ordering the components in different subjects in the same way and produces a single set of components that can then be more easily interpreted. The number of time points of the fMRI data has no relationship to the number of independent sources. There are 220 points in the timeseries, much more than the number of independent sources in the fMRI data. Typically, principal component analysis (PCA) is used to represent most of the variance of the data while drastically lowering the dimensionality6. We used two data reduction steps, one on data from each subject (reduction from 180 to 60 components) and one on an aggregate data set (reduction from 1200 to 40 components). Within each reduction step, the data variance was largely preserved, more than 99%. The group level ICA results in 40 independent components calculated using the Infomax algorithm7. The components were transformed in Z-score maps and thresholded at Z > 3.1, allowing to evidence spatial patterns of highly connected areas. The maps were visually inspected to identify the Default Mode Network (DMN) and the network encompassing the Orbitofrontal regions. The two networks under study are shown in Figure 2 and Figure 3. One may observe that they mostly correspond to two well-documented intrinsic connectivity networks evidenced during rest (doi:10.1162/jocn_a_00077; Figure 2, network 13 and network 2).
